# Interrelationships between the extracellular matrix and the immune microenvironment that govern epithelial tumour progression

**DOI:** 10.1042/CS20210679

**Published:** 2022-03-09

**Authors:** Natasha Kolesnikoff, Chun-Hsien Chen, Michael Susithiran Samuel

**Affiliations:** 1 Centre for Cancer Biology, SA Pathology and University of South Australia, Adelaide, South Australia, Australia; 2 Adelaide Medical School, Faculty of Health Sciences, University of Adelaide, Adelaide, Australia

**Keywords:** Extracellular matrix, infiltration, stroma, tumour immunity, wound healing

## Abstract

Solid tumours are composed of cancer cells characterised by genetic mutations that underpin the disease, but also contain a suite of genetically normal cells and the extracellular matrix (ECM). These two latter components are constituents of the tumour microenvironment (TME), and are key determinants of tumour biology and thereby the outcomes for patients. The tumour ECM has been the subject of intense research over the past two decades, revealing key biochemical and mechanobiological principles that underpin its role in tumour cell proliferation and survival. However, the ECM also strongly influences the genetically normal immune cells within the microenvironment, regulating not only their proliferation and survival, but also their differentiation and access to tumour cells. Here we review recent advances in our knowledge of how the ECM regulates the tumour immune microenvironment and *vice versa*, comparing normal skin wound healing to the pathological condition of tumour progression.

## Introduction

Tumours consist not only of neoplastic cells but also an altered stroma or microenvironment that is distinct to that found in the corresponding normal tissues. The tumour microenvironment (TME) describes constituents of the tumour mass that are not tumour cells, and is a key focus of research because it contributes to tumorigenesis, invasion and metastasis, as well as to resistance to chemotherapy and radiotherapy. The TME comprises both cellular and non-cellular components. The cellular portion includes diverse stromal cells, including cancer-associated fibroblasts (CAFs), immune cells (tumour-associated macrophages [TAMs], lymphocytes, mast cells [MCs]), endothelial cells, pericytes, adipocytes, nerve cells and others [1,2]. A key feature of these cells is that they have not undergone oncogenic transformation. The non-cellular portion of the TME is the extracellular matrix (ECM), a protein scaffold that provides architectural and mechanical support, anchorage for cell adhesion, storage of water and various growth factors, and induces intracellular signaling pathways via cell–ECM interactions [3]. CAFs are among the most abundant cell types in the microenvironment of solid tumours, second only to tumour cells themselves. CAFs are activated or re-educated mesenchymal-like cells in the TME and exhibit considerable cellular heterogeneity. CAFs can exhibit tumour-promoting functions or tumour-suppressive capability depending on their particular context, and act by releasing various growth factors (such as epidermal growth factors [EGFs], transforming growth factor [TGF]-β and hepatocyte growth factor [HGF]) and an array of chemokines to induce different aspects of tumour cell behaviour [4]. CAFs also regulate tumour cell behaviour by regulating mechanical cues exerted by the ECM [5]. CAFs have an important role in regulating immune cell behaviour within the TME, including potentially their differentiation and identity [6], thereby modulating the ability of the immune system to find and destroy cancer cells [7].

Immune surveillance is compromised in many tumours [8–10]. Cancer cells evade immune destruction by polarising infiltrating immune cells such as macrophages and lymphocytes into tumour promoting or immunosuppressive states, depending on the context. Therefore, a tumour can be viewed not as a collection of relatively homogeneous cancer cells but instead as a novel organ in which cancer cells interact with different specialised stromal cells and ECM that operate together in a coordinated fashion [6]. By investigating and further understanding the acquired capabilities necessary for tumour growth and progression, measures may be taken, or interventions developed to target the specific mechanisms contributing to cancer progression. Here, we discuss key similarities between the microenvironments of epithelial cancers and healing wounds to leverage our existing knowledge of wound healing to elucidate the tumour microenvironment.

## The microenvironment in skin wound healing and tumorigenesis

**Figure 1:**
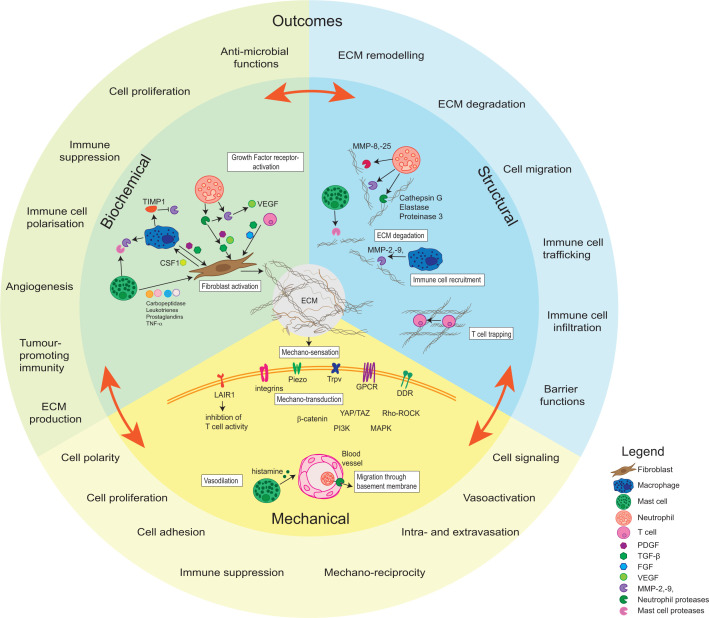
Biochemical, structural and mechanical interrelationships between the ECM and tumour-associated immune cells determine key disease outcomes The ECM is largely produced, remodelled and degraded by a suite of genetically normal cell types within the microenvironment. This figure highlights key relationships between immune cells, CAFs and the ECM that regulate the biochemical, structural and mechanical characteristics of the ECM, which in turn regulate the cellular functions determining the course of the disease.

Tissue homoeostasis is a meticulously regulated and dynamic process that occurs throughout the lifetime of an organism including during development, and can be perturbed during pathological processes such as wound healing and tumorigenesis (Figure 1).

Disruption of skin homoeostasis by wounding triggers a cascade of events requiring the interaction of local keratinocytes, fibroblasts of the connective tissue, and the immune system, aimed at restoring skin integrity. The first step of wound healing is haemostasis, during which the body responds to the injury and coagulation of blood stops leakage from damaged vessels. From this stage, the platelets begin restoration of tissue structure by stimulating angiogenesis and fibroblast activation, but also inflammation by recruiting neutrophils and monocytes. The inflammation and proliferation phases require the interaction of immune cells and connective tissue fibroblasts to clear debris and infection and restore the ECM. Wound healing is complete in the maturation phase where re-establishment of the connective tissue and re-epithelialisation are completed, and homoeostasis is restored [11].

In the early phase of wound healing, the provisional ECM deposited by macrophages and activated fibroblasts is a disorganised matrix consisting predominantly of fibrin and fibronectin (FN), which is subsequently replaced by the normal ECM, composed of collagen and glycoproteins, prior to resolution. The composition of the ECM that is established may influence the type and extent of inflammation that occurs, with scarless healing characterised by low levels of inflammation and scarring characterised by high levels of inflammation [12]. While it is still unclear which of these are cause and consequence, the clear association between these two phenomena is striking.

Similar molecular mechanisms and cellular behaviour are co-opted during tumorigenesis, except that wound healing is a finely tuned and self-limiting process; whereas tumorigenesis is, in contrast, characterised by dysregulated cellular behaviour and signalling pathways, which while being well-coordinated, are not self-limiting [13]. The similarities between the two processes have been appreciated for some time, since Dvorak postulated the idea that tumours are wounds that do not heal [14]. Like the wound healing microenvironment, the TME contains fibroblasts, immune cells (lymphocytes, macrophages, MCs), other stromal cells (endothelial cells, adipocytes, pericytes) as well as ECM.

In the acute inflammation response to wound healing, the first two immune cell types recruited into and surrounding the wound are neutrophils and monocytes, which differentiate into macrophages. Both neutrophils and macrophages boost inflammatory responses by secreting pro-inflammatory cytokines such as tumour necrosis factor (TNF)-α, interleukin (IL)-1β and IL-6 [15]. Macrophages exist on a phenotypic continuum, ranging between the pro-inflammatory (M1) and anti-inflammatory (M2) polarised states. In the early phase of wound healing, expression both M1 and M2 markers are observed, while later in wound healing, macrophages exhibiting M2 markers predominate [16]. Intriguingly, the condition of chronic inflammation has long been associated with a microenvironment conducive for tumorigenesis and cancer progression [17,18]. TAMs are one of the main tumour-infiltrating immune cell types that participate in the formation of the TME. It has been demonstrated that M2 TAMs in TME promote tumour cell proliferation, invasion/metastasis and angiogenesis through their secretion of cytokines (IL-10, IL-1β), chemokines and growth factors (vascular endothelial growth factor [VEGF], TGF-β), as well as ECM remodelling proteases including matrix metalloproteinases (MMPs). In a manner similar to that proposed for wounds above, the properties of the tumour ECM strongly influence the type and extent of inflammation that occurs within the tumour.

## The ECM in wound healing and tumorigenesis

The ECM is the acellular constituent of all tissues and organs of the body and is also a key component of tumours [19]. It consists not only of a wide range of macromolecules that form a three-dimensional fibrillar architecture that surrounds and supports cells but also serves as a reservoir of cytokines, chemokines, and growth factors that modulate cell fate. Furthermore, the ECM plays a significant role both in normal physiological homoeostasis and in pathological process such as inflammation, fibrosis and tumorigenesis. The tumour ECM forms a scaffold within which tumour cells and the cellular constituents of the TME exist. In cancers, it is well documented that the ECM has both anti-tumorigenic and pro-tumorigenic functions, including the potential to foster or indeed suppress cancer initiation, cancer cell migration/invasion, metastatic dissemination and distant colonisation. The ECM of tumours is commonly dysregulated and disorganised relative to the ECM of the corresponding normal tissue, and implicated in eliciting aberrant cellular behaviour in both cancer cells and stromal cells, thereby promoting cellular transformation and metastasis [20].

## Maintenance of the ECM in normal tissue homoeostasis

The ECM is synthesised by all cell types to varying amounts, but the main producers of ECM are cells of the connective tissue, including stromal cells such as fibroblasts, immune cells, and pericytes. Epithelial, neuronal, and muscle cells may also produce ECM depending on the state of the tissues they populate – for instance, whether the tissue is in the process of development, healing following injury, fibrotic or in a state of tumorigenesis [19,21]. Production of ECM by these cells is governed by a suite of signalling pathways generally termed mechanotransduction pathways, which are regulated by feedback from key properties of the ECM itself, a phenomenon termed mechanoreciprocity, but that may also be engaged by classical signalling molecules such as growth factors and cytokines [5,22].

Mechanotransduction pathways can be initiated by several means including by mechanically induced integrin clustering [23], engagement of discoidin domain receptor (DDR) tyrosine kinases or activation of mechanically regulated ion channels in a variety of cell types [24], and the leucocyte-associated immunoglobulin-like receptor 1 (LAIR1/CD305) in white blood cells [25]. Downstream of these mechanical inputs, signalling pathways including those mediated by mitogen-activated protein kinases (MAPKs), Rho-associated kinase (ROCK), Phosphoinositide 3-kinase (PI3K) and Yes-associated protein (YAP) and its homologues the TAZ proteins are key mechanotransduction pathways that convert mechanical signals into functional biochemical and mechanical outputs. For example, signalling through ROCK results in enhanced actomyosin contractility, thereby permitting cells to respond to external mechanical stresses, but also regulates the production and remodelling of ECM proteins within tissues in normal tissue homoeostasis, wound healing and tumorigenesis [26–29]. A similar mechanism is also regulated by YAP signalling [30,31], suggesting a principle of reciprocal regulation of intracellular and extracellular mechanical stresses [32] exerted by the cellular cytoskeleton and the ECM respectively.

## Components of the ECM

The entire set of genes coding the matrix and matrix-associated proteins is termed the ‘matrisome’ [33]. In human and mouse genomes, there are approximately 1050 matrix genes, which can be categorised into those encoding core-matrisome proteins (∼280 genes) and matrisome-associated proteins (∼780 genes). The core-matrisome proteins are a collection of ECM glycoproteins, collagens and proteoglycans. The matrisome-associated proteins comprise ECM-affiliated proteins, ECM regulators and secreted factors. Using human tumour xenografts in mice and proteomic analysis, Naba et al. demonstrated that the tumour ECM is contributed by both tumour cells and stromal cells [33]. Tumour cells with varying metastatic potential secrete distinct suites of matrisome components. Furthermore, the stromal cell-secreted matrisome components differ according to the metastatic potential of the tumour cells, indicating cross-talk between the tumour cells and the stromal cells [33].

Collagens, as the main structural element of the matrix, are generally the most abundant protein within the ECM, and indeed throughout the body. At least 28 different types of collagens have been identified in vertebrates. Collagens provide mechanical strength and bioactive sites for cell adhesion. Fibrillar collagens, such as collagen type I, are the major component of tumour desmoplasia and are critical players in tumour cell survival and metastasis in many tumour types, such as breast cancers [26,34], pancreatic ductal adenocarcinoma [28,35] and skin tumours [27]. Compared with the benign lesions in which type I and type III collagen bundles are regularly organised, malignant human breast tumours show high expression of type I and type III collagen and newly synthesised collagens are frequently arranged in abnormal bundles, particularly at the invasive front of malignant tumours. Fibroblast-like cells of the stroma rather than malignant epithelial cells contribute most to the high level of collagen within the tumour ECM. The aberrant and destructive pattern of collagen bundles suggests a more readily degradable TME and thus may facilitate breast tumour invasion [36]. Network-forming collagens are rich in basement membrane which define boundaries within tissues and separate epithelial or endothelial layers from the interstitial matrix and are essential for tissue polarity. It is well established that the basement membrane must be breached or traversed by cancer cells prior to invasion, metastasis and tumour angiogenesis [37–39]. MMPs, which are divided into secreted and membrane-bound MMPs are the major family of matrix-degrading proteolytic enzymes. MMPs cleave a wide range of substrates, such as collagen, FN, laminin and elastin (ELN) and involve in degradation of basement membranes and other ECM barriers, participating in both normal processes and pathogenesis noted in cancer progression [40]. By substrate specificity and cellular localisation, MMPs can be divided into collagenases (MMP-1, 8 and 13), gelatinases (MMP-2 and 9), stromelysins (MMP-3, 10 and 11), matrilysins (MMP-7 and 26) and membrane-type MT-MMPs (MMP-14, 15, 16 and 17) [41]. Of note, membrane type 1-MMP (MT1-MMP; also designated MMP-14) has important roles in matrix turnover and regulates key functional characteristics of breast cancer cells, such as migration, invasion and angiogenesis [41]. Up-regulation of active MT1-MMP has been noted in CAFs of the TME in mouse models of breast cancer [42]. MT1-MMP-mediated proteolysis by stromal cells is important in the metastatic process in mammary cancers of PyMT mice [43]. *MMP14* gene expression is remarkably higher in tissues from invasive breast carcinoma compared with normal tissues. High *MMP14* expression is related to poor survival in ERα-negative breast cancer and triple-negative breast cancer patients [41].

The other two groups of the core matrisome are glycoproteins and proteoglycans. Glycoproteins are a group of proteins with short, branched oligosaccharide chains covalently attached to a central polypeptide core. Proteoglycans are glycoproteins with attached glycosaminoglycans (GAGs) which confer on proteoglycans a high negative charge and the ability to store water and divalent cations such as calcium [44], and have been implicated in CAF education [45]. GAGs are long, linear, acidic carbohydrate polymers with repeating disaccharides comprising heparan sulphate/heparin (HS/HP), chondroitin sulphate/dermatan sulphate (CS/DS), keratan sulphate (KS) and hyaluronic acid (HA). Heparan sulphates on proteoglycans bind many secreted and growth factors into the ECM [46]. Dysregulation of glycoproteins including laminins, FNs, tenascins, and periostin (POSTN) has been observed in the TME. Laminins together with collagen IV are primary components of the basement membrane that provide a structural barrier to cancer cell invasion, intravasation, and extravasation [47]. The glycocalyx, mainly composed of proteoglycans and glycoproteins, is a surface layer that covers all cells, including cancer cells. The thickness of the glycocalyx on cancer cells is related to integrin-mediated cellular adhesion to ECM, cell growth, and cell survival [48]. A thick glycocalyx is frequently observed on cancer cells. By expression of large tumour-associated glycoproteins such as MUC1, a bulky glycocalyx on non-transformed mammary epithelial cells promoted focal adhesion assembly and facilitated integrin-dependent ERK and AKT signalling to support cell growth and survival. Large glycoproteins (such as MUC1 and CD44) are abundantly expressed on circulating tumour cells isolated from patients with metastatic breast cancer that indicated a bulky glycocalyx on tumour cells could favour tumour cell dissemination and metastasis [48].

FNs have the ability to bind simultaneously to cell surface receptors, collagen, proteoglycans, and other FN molecules providing essential ECM–cellular connections to cells through cell surface integrin receptors and other receptors to regulate cell adhesion, migration, and differentiation [49]. FN polymerisation into the ECM is required for the production of collagen-I and thrombospondin (TSP)-1 indicating that FN polymerisation is a critical regulator of ECM organisation and stability [50]. In cancer, FN is expressed by both CAFs and cancer cells. Alignment of the FN fibres is a prominent feature of both prostatic and pancreatic cancer stromata, in which FNs assembled by CAFs are deposited in an anisotropic manner, which guide cancer cells to migrate via αv integrin engagement on the surface of prostate cancer cells [51].

ELN is a fibrillar protein with highly elastic characteristics expressed in tissues that need the ability to resume their shape following stretching. As such it is a key constituent of the dermis and has the unusual characteristics of being a very long lived protein that is minimally turned over in health [52]. As such, adult wound healing does not regenerate the full ELN composition of unwounded skin, resulting in scars that lack elasticity. In contrast, one of the processes that establishes the tumour ECM is the production of ELN, termed tumour elastosis, which has been associated with both tumour-promotion and tumour-suppression [53]. These observations suggest context-dependent functions, possibly associated with the capacity peptides produced from ELN degradation to interact with the elastin receptor complex (ERC) [54] and other receptors such as integrins, as well as the ability of lysyl oxidase to cross-link ELN fibres with collagen fibres [55]. The ERC contains neuraminidase 1, which is activated upon ligand engagement with the ERC, causing cell type-dependent activation of effector pathways, including the tumour-promoting MAPK signalling pathway [56]. Inhibition of ELN degradation, lysyl oxidases and the ERC are therefore worth exploring as potential therapeutic approaches to both enhance scarless healing and suppress tumour progression. Tenascin-C (TNC), expressed by both transformed epithelial cells and stromal cells in the TME, is up-regulated in cancerous tissue and strongly associated with invasiveness and metastasis [57]. In breast cancer, the expression of TNC is correlated with the aggressiveness of pulmonary metastasis. TNC enhances the WNT pathway and thereby cancer cell proliferation, via its positive regulator Musashi homologue 1 (MSI1) and NOTCH signalling in breast cancer cells [58]. TNC produced by metastasis-initiating breast cancer cells is critical for metastasis outgrowth in the pulmonary parenchyma, whereupon infiltrating myofibroblasts or another stromal cells take over as a source of TNC to facilitate subsequent growth [58]. It is notable that TNC is also implicated in chemoresistance; in xenograft mouse models of breast cancer, exposure to multiple chemotherapy agents was shown to activate the JNK pathway which enhances the expression of secreted phosphoprotein 1 (SPP1) and TNC via c-Jun transcription factor, leading to reduced therapeutic efficacy and progression of lung metastasis. Inhibition of JNK signalling or down-regulation of SPP1 or TNC can sensitise mammary tumours to chemotherapy and suppress the progression of metastatic breast cancer [59].

POSTN is highly expressed, mainly by stromal cells such as CAFs and to a lesser extent by tumour cells, and this is frequently associated with poor prognosis and metastasis in solid tumours. POSTN interacts with integrins expressed on the surface of tumour cells and initiates numerous signalling pathways that regulate cell proliferation, cell survival, or cell migration [60]. In oral squamous cell carcinoma (OSCC), overexpression of POSTN is frequently observed and is correlated with the pattern of invasion and metastasis. POSTN-positive cases of OSCC had higher blood vessel density and recombinant POSTN enhanced capillary formation *in vitro* in a concentration-dependent manner which suggests that POSTN may promote angiogenesis [61]. In pancreatic ductal adenocarcinoma, POSTN interacts with α6β4 integrin in cancer cells leading to phosphorylation of FAK and AKT through activation of the PI3K pathway and promoting the invasiveness of tumour cells by increasing the motility of cells [62]. In head and neck squamous cell carcinoma, POSTN secreted by CAFs might promote cancer stemness through interacting with protein tyrosine kinase 7-Wnt/β-catenin signalling pathway in cancer cells [63]. In the MMTV-PyMT mouse breast cancer model, infiltrating cancer cells need to induce stromal POSTN expression to initiate lung metastatic colonisation. The production of POSTN then augments Wnt signalling to facilitate cancer stem cell maintenance and promote metastasis [64].

The foregoing highlights a strong pattern of ECM involvement with tumour progression by direct engagement of mechanotransduction pathways by ECM components. However, another way in which the ECM influences disease progression is via its effects on the tumour immune system, which will be discussed in the next several sections.

## The immune microenvironment in normal homoeostasis

The immune microenvironment is strongly influenced by characteristics of the ECM, while also regulating its production and remodelling (Figure 1). Many immune cell types engage directly with the ECM, which defines their biology and function. Furthermore, different immune cell types have key roles in ECM homoeostasis, participating in the production of ECM components, structurally remodelling the ECM and degrading it as required.

### Inflammation in skin wound healing and the TME

Inflammation during cutaneous wound healing serves key functions beyond host defence against invading pathogens. The primary immune cell players which include neutrophils, monocyte–macrophages, MCs, and T lymphocytes also produce cytokines, chemokines, growth factors, and proteases that support proliferation of the epithelial cells as well as production and remodelling of ECM by fibroblasts [65]. Fibroblasts of the connective tissue are critical for the restoration of the ECM during wound healing, but also have important interactions with the immune system.

Cross-talk between the immune system and fibroblasts of the connective tissue is critical for efficient wound healing. Disruption of this interplay can lead to a range of outcomes from relatively minor aberrations such as scar formation to severe problems such as fibrosis. The outcomes of the wound healing process therefore rely on the precisely regulated interactions between all cell types involved as well as with the ECM. However, the process that these immune cells play in wound healing can be hijacked to establish a pro-tumorigenic microenvironment, in which a cancer develops as the well-canvassed ‘wound that never heals’ [14]. In this section, we review the major players of the immune system during wound healing, and how they modulate the ECM, to draw lessons on how these cells may be co-opted to establish and maintain the TME.

## Constituents of the immune microenvironment and their interactions with the ECM

Immune cell recruitment is initiated by signalling from damage-associated molecular patterns (DAMPs), pathogen-associated molecular patterns (PAMPs), and platelets, which mediate coagulation. Immune cells responding early include neutrophils and monocyte–macrophages, as well as tissue-resident MCs. These cell types all play critical roles in host protection, as well as activation of fibroblasts and keratinocytes. While T lymphocytes are also recruited, they are not critical for the wound healing processes. In homoeostasis, key immune functions are performed by tissue-resident immune cells, but during wound healing the primary active players are recruited via the vasculature. Mediators released by resident MCs can aid in permeability of the vasculature, but neutrophils in particular can transmigrate with very little remodelling of the vessels [66]. Recruitment of the immune cell players into the wound site is reliant on biochemical signaling, but also requires mechanical and structural alterations to allow passage of the cells. ECM collagen structure, organisation, and matrix pore size can also therefore influence immune cell infiltration [67,68]. Therefore, migration of immune cells through the connective tissue is dependent on MMP-mediated ECM degradation to enlarge pore diameter, and integrin- and actomyosin-dependent exertion of force for path generation.

What follows is a brief description of each immune cell type, how they are recruited and the functions they fulfil in wound healing and tumour progression. We also discuss factors expressed by the cell that can influence ECM structure, and mechanisms by which they interact with fibroblasts as the major ECM producers. In the final section, we discuss how these features of the immune cells act to either promote or suppress tumorigenesis.

### The ECM in neutrophil recruitment and function

Neutrophils are recruited to the site of tissue injury by DAMPs also called Alarmins, which include molecules such as DNA and high mobility group box protein (HMGB)-1, which are referred to as nuclear DAMPs, ATP and uric acid, termed cytosolic DAMPs and components of the ECM [69]. These signals are produced by damaged keratinocytes and neutrophils are typically the first immune cells to respond to them [70]. Sustained neutrophil recruitment is mediated by chemokines and lipid mediators such as CXCL8/IL-8 family and leukotriene B4 (LTB4), which are also DAMP-activated and released from endothelial cells [71]. IL-8 (aka CXCL8) is a well-known chemoattractant for neutrophils and signals via the receptors CXCR1 and CXCR2. CXCR2-deficient mice have impaired cutaneous wound healing [72], highlighting the importance of this chemokine in the skin.

In conjunction with their roles as innate defenders against pathogens, neutrophils can also produce pro-inflammatory cytokines and chemokines, promote angiogenesis, degrade the ECM, and influence the migration, proliferation and activation of keratinocytes and fibroblasts [73]. Once spent, neutrophils undergo apoptosis and are cleared by macrophages. This initiates a feed forward pro-resolution programme that is characterised by the release of the tissue repairing cytokines, TGF-β and IL-10.

These functions are facilitated by degranulation, which is the release of pre-formed and stored cytoplasmic granules, providing the materials for a quick response during tissue injury. There are four main types of granules – azurophilic (or primary) granules, specific (or secondary) granules, gelatinase (or tertiary) granules, and secretory vesicles (SVs) – each of which differs in its contents, signals required for granule secretion, and site of secretion [74]. In settings of infection and inflammation neutrophil extracellular traps (NETs) and extracellular vesicle exosomes [75] are also important sources for ECM-degrading proteases.

A significant component of primary granules are the serine proteases, of which elastase, proteinase 3, and cathepsin G are major components [76]. Neutrophil extravasation to site of injury can be minimally disruptive to vasculature. Migration through the vessel basement membrane, the part of the ECM first encountered by extravasating neutrophils, is facilitated by neutrophil-elastase and by passing through gaps in the pericytes where there are reduced levels of the ECM proteins laminin 10, collagen IV, and nidogen-2 [66]. In settings of disease and inflammation, the neutrophil serine proteases function to ingest pathogens, as well as to break down ECM to aid in migration [76]. Degradative targets within ECM include ELN, FN, laminin, and collagen IV [76]. Moreover, the serine proteases can promote MMP activity by cleavage of the pro-form of these enzymes to engage their catalytic activity [77].

The secondary and tertiary particles of neutrophils contain many matrix-degrading enzymes. Neutrophil-sourced MMP-8 can degrade collagen I and III, MMP-9 can cleave collagen IV [78], and MMP-25 can digest collagen IV and FN [79]. During wound healing, degradation of the ECM occurs to facilitate neo-angiogenesis within the restored tissue, which is key to normal regeneration. Neutrophils are a source of VEGF, a major growth factor that can stimulate angiogenesis [80] but can also cause epithelial cells to produce VEGF. Other pro-angiogenic factors such as MMPs, can degrade ECM and release ECM-bound VEGF which is then free to initiate signalling. Neutrophils are the only cells that can release MMP-9 free of its endogenous inhibitor, tissue inhibitor of metalloproteinases (TIMPs), and can directly deliver MMP-9 to sites of angiogenesis [81].

Neutrophils have also been known to activate fibroblasts during wound healing. During healing after myocardial infarction, neutrophils aid fibroblast in producing the provisional fibrin/fibronectin matrix, and stimulate fibroblast differentiation, collagen synthesis, and macrophage polarisation [82]. Formation of NETs induces activation of fibroblasts and differentiation into myofibroblast phenotype, which had increased connective tissue growth factor expression, collagen production, and proliferation/migration [83].

The very diverse functions of neutrophils in wound healing therefore requires a balance of factors to orchestrate migration, inflammation, angiogenesis, and fibroblast activation to produce effective healing. The opportunity for these same characteristics to be co-opted to detrimental effect in tumorigenesis are striking.

### Tumour-associated neutrophils

Neutrophils are attracted to tumours by tumour-derived IL-1β, prostaglandin E2 (PGE2), and IL-8 [84]. In the early stages of tumorigenesis, as in wound healing, tumour-associated neutrophils (TANs) behave in a cytotoxic manner, producing high levels of TNF-α, NO, and H_2_O_2_ [85]. At later stages of tumorigenesis, neutrophils can exhibit a tumour-promoting phenotype. This change in function is associated with reduced prognostic value and a high incidence of metastasis [86]. Neutrophil polarisation is driven by TGF-β [87], and TANs are thought to exist within a continuum of states bounded by the pro-tumour N1 and anti-tumour N2 states [87].

The actions of tumour-promoting TANs include enhancement of angiogenesis, tumour cell proliferation, and immuno-suppression. TANs can suppress T-cell function in colorectal cancer (CRC) by secreting MMPs to activate TGF-β-mediated T-cell suppression [88]. TANs drive tumour angiogenesis by a range of mechanisms, including activation of pro-MMP-2 by elastase, cathepsin G, and proteinase-3 [77]. Neutrophil elastase and MMP-9 can each support angiogenesis by activation of VEGF [89,90]. Elastase from TANs can in fact activate latent forms of VEGF, platelet-derived growth factor (PDGF), and TGF-β to drive tumour cell proliferation and migration [90,91], but may also function via its role in regulating ECM homoeostasis [92].

ECM remodelling plays a role in the cellular mechanism underpinning NET-induced reversal of tumour cell dormancy and the resumption of tumour cell proliferation. NET-associated proteases neutrophil elastase and MMP-9 can induce re-awakening of dormant cancer cells through ECM remodelling cleaving of laminin-111, leading to activation of integrin a3β1 [93] and downstream signalling molecules including FAK, ROCK, YAP and MEK, resulting in tumour recurrence, disease relapse, and metastasis [93].

### Macrophages in tissue and ECM homoeostasis

Macrophages are indispensable for cutaneous wound healing [94,95]. Monocytes are recruited to the wound site by a range of cytokine signals, and are terminally differentiated *in situ* into macrophages and dendritic cells. Chemoattractants for monocytes in wound healing include CCL-2 (MCP-1), CCL-3 (MIP1α), CCL-4 (MIP-1β), CCL-5, TSP-1, IL-1, IL-6, and TNF-α [96,97]. Neutrophils, which secrete many of these factors, can therefore recruit monocytes to the wound, and the mature macrophages then clear spent neutrophils from the wound site via phagocytosis.

Wound macrophages display heterogeneous phenotypes usually described as a continuum bound by the M1 and M2 phenotypic states [98]. At the initial stages of healing, most macrophages are polarised towards a pro-inflammatory M1 phenotypic state which aids in the clearance of pathogens, dead neutrophils, and dead tissue. After this initial phase, macrophages transition to a largely M2 anti-inflammatory phenotype and encourage re-epithelialisation, stimulate fibroblast migration and proliferation, and angiogenesis [95]. At the final phase of wound healing, macrophages release MMPs to digest the provisional ECM and then undergo apoptosis so that the skin can return to homoeostasis. In chronic wounds, macrophages retain their pro-inflammatory phenotype, resulting in persistent inflammation that impedes tissue repair.

Macrophage polarisation towards a more M1 state is mediated by interferon (IFN)-γ, TNF-α and GM-CSF, and can be identified by surface markers CD68, CD80 and CD86, and the secretion of pro-inflammatory cytokines such as IL-1β, IL-6, IL-12, IL-23, TNF-α, CXCL-9, and CXCL-10. Whereas IL-4, IL-13, IL-10, and TGF-β induce the M2 phenotype, which is characterised by the expression of CD204, CD206, and CD163 surface markers, and the secretion of anti-inflammatory cytokines such as IL-10, TGF-β, CCL-17, CCL-18, CCL-22, and CCL-24 [99]. Midway through healing, macrophages expressing CD301b produce high levels of IL-10, PDGF-β, and TGF-β [100] – these factors are necessary for efficient re-epithelisation, revascularisation, and fibroblast regeneration.

Macrophages are an important source of MMP-2 and MMP-9 to degrade ECM [101]. However, unlike neutrophil-released MMP-9, macrophage-produced MMP-9 is bound by TIMP1, which limits MMP-9 activity [81]. Therefore, macrophages appear to have an intrinsic modulatory mechanism for MMP function. MMPs and the role of macrophages in modulating their activity are important for the remodelling phase of wound healing to alter the composition of the nascent ECM and to re-establish normal ECM within the healed skin [98].

A key role of macrophages during wound healing is the mutual regulation of other cells of the microenvironment. Fibroblasts secrete CSF1, an essential growth factor for macrophages, and macrophages can conversely stimulate fibroblast proliferation by producing PDGFα and activated macrophages can enhance ECM hyaluronan and ELN synthesis by fibroblasts [102]. Neutrophil recruitment of monocytes induces their own phagocytosis and this CD18 (integrin β2)-dependent contact can stimulate the expression of TGF-β in macrophages [103]. TGF-β is a major cytokine that induces fibroblast differentiation into myofibroblasts, with concomitant α-SMA expression, and wound contraction resulting from the ability of these cells to physically contract the ECM [104,105].

Conversely, fibroblasts are also able to recruit macrophages. This can be by biochemical means, via fibroblast-secreted TSP-1 [97] or by physical means via fibroblasts-mediated construction of ECM tunnels that modulate macrophage migration [106] and the mechanosensation by macrophages of contractile forces exerted by myofibroblast upon the ECM [107], although this latter mechanism has only so far been demonstrated *in vitro*.

### TAMs and the ECM

Polarisation of macrophages during cutaneous wound healing is critical for the switch from pro-inflammatory to anti-inflammatory and pro-re-epithelisation phases. However these characteristics of macrophages also contribute to their roles in tumorigenesis. As such, macrophages polarised towards the M1 phenotypic state are associated with tumour-suppressive functions such as supporting CD8^+^ cytotoxic T-cell activity. Macrophages polarised towards the M2 state are anti-inflammatory and associated with tumour-promoting functions such as ECM remodelling, angiogenesis, stimulating cancer cell proliferation and metastasis. Functions of macrophages that promote wound healing, particularly in the later phases of the process, are favourable for tumour progression and often co-opted into the process.

The role of TAMs is perhaps best studied in the context of their interaction with CAFs. TAMs and CAFs can induce activation and recruitment of each other, in a manner similar to that observed in normal macrophages and tissue fibroblasts during wound healing. CAFs release fibroblast growth factors that induce M2 polarity in macrophages. M2 cells release TGF-β causing fibroblast reprogramming to a tumour-promoting CAF state. It has been shown using an orthotopic CRC model, that recruitment of TAMs to the tumour site regulated a suite of ECM-associated genes which established key characteristics of the TME. Many of these ECM components were expressed by the TAMs directly, but others were produced by CAFs under the influence of TAMs [108].

One mechanism by which tumour-suppressing TAMs inhibit tumour growth is by remodelling tumour ECM to destroy the physical barrier that inhibits the recruitment of cytotoxic T cells, and thereby enhancing their access to the tumour. This mechanism has been harnessed for chimeric antigen receptor (CAR) macrophage cell therapy in a proof of principle study by engineering macrophages to express the CD147 antigen, a cell surface protein of the immunoglobulin superfamily with the ability to enhance the production of MMPs by an as yet imprecisely defined mechanism. CAR-147 macrophages expressing the CD147 antigen reduced ECM density and collagen content by increasing the production of MMP-3, MMP-14, and MMP-15, thereby facilitating the entry of cytotoxic T cells into 4T1 cell allografts in immune-competent mice [109].

### MCs and their modulation of inflammation

MCs are key effector cells of the innate immune system whose principal function is to aid in host defence against pathogens. Present in almost all tissues in the body, they are largely located where the organism interfaces with the environment such in the skin, gastrointestinal tract, and respiratory tracts. Upon activation, MCs rapidly degranulate and release their protein-packed granules that contain pre-made chemokines, cytokines growth factors, histamine, and proteases. They are also capable of *de novo* synthesis of a suite of other proteins that are also released following activation [110].

MC activity is required for proper wound healing [111], as well as to restrain scar ECM formation via the deployment of proteases [112,113]. Functionally, MCs exert a protective effect by directly targeting pathogens, but also recruit and activate neutrophils, macrophages, and T lymphocytes [114,115]. The release of histamine can facilitate vasoactivation [116], and pro-inflammatory cytokines IL-6 and IL-8, and VEGF can recruit other immune cells [117]. Additionally, MCs can contribute to the polarisation of macrophages in wounds [118]. The MC-specific proteases chymase and tryptase are also released during early inflammation and serve to break down the ECM [119]. But conversely, MCs can also modulate fibroblast activation [120] and ECM production.

MCs are recruited to the wound site by chemokines such as CCL-2 and CCL-5, as well as growth factors such as stem cell factor (SCF). Membrane-bound SCF on dermal fibroblasts can induce MC progenitor maturation by binding to surface c-kit [121]. The SCF stimulation can also aid in the adherence of MCs to FN [122]. Thrombin has also been found to stabilise MC adhesion to FN and laminin, in addition to activation to release cytokines and proteases [123]. Indeed, the attachment to FN has been shown to stabilise MCs and modulate pro-inflammatory cytokine release [124,125].

MCs have roles in ECM production, processing and degradation. MC proteases can degrade collagen [126], FN [127], laminin [112,113] and activate the latent forms of MMP-2 and MMP-9 [128]. MC produced prostaglandin D2 and leukotriene D4, carbopeptidase A, and tryptase can stimulate dermal fibroblast collagen production and proliferation [129]. MC chymase can activate endothelin-1, which in turn can induce myofibroblast differentiation and collagen synthesis in dermal fibroblasts through the RhoA/Rho kinase pathways [130]

In the settings of infection, MCs can also recruit neutrophils by producing TNF [131], tryptase [132], and leukotrienes, LTB4 and LTC4 [133].

### MC-mediated regulation of the tumour ECM

MCs accumulate in tumour margins in proximity to vessels. In early tumours, it is thought that MCs are pro-inflammatory and recruit cells to support anti-tumour functions. However during later stages MCs mediate angiogenesis and metastasis. The observed heterogeneity in the functions of MCs in tumour settings, and the specific consequences of MC accumulation likely depends on tumour type and grade [134].

As in normal tissue, MCs in tumours can produce both pro- and anti-inflammatory cytokines and chemokines, growth factors to stimulate angiogenesis, and proteinases to remodel the ECM. MC proteases chymase and tryptase can stimulate angiogenesis in cutaneous carcinoma, and tryptase has mitogenic effect on dermal fibroblasts. Additionally, these proteases can activate the pro-forms of MMP-2 and MMP-9 to facilitate ECM remodelling [135]. However, the precise conditions under which MCs exhibit pro- vs. anti-tumour effects upon the ECM and the suite of immune cells with which they cooperate in order to exert these effects still remain somewhat ill-defined. Nevertheless, the accumulation of MC at tumour margins suggests a role for these enigmatic cells in tumorigenesis.

### T cells in tissue homoeostasis and cancer

T lymphocytes are not crucial for effective wound healing; however they can contribute to each relevant process by secreting cytokines and growth factors to regulate immune response and fibroblast activation.

In an murine excisional wounding model, it was observed that CD4^+^ T cells are the predominant subtype recruited into the wound [136]. This time-course study by Chen et al. demonstrated that CD4^+^ and CD8^+^ T cells migrated into cutaneous wounds, with CD4^+^ T cells present from day 1 (peak numbers were observed at day 5 and had dropped by day 10), and CD8^+^ cells recruited from day 3 (peak numbers observed at day 7 and had dropped by day 21). On day 7, CD4^+^ T cells expressed IFN-γ, TGF-β, IL-10, and IL-17 at high levels (relative to their naïve counterparts). CD8^+^ T cells had high IFN-γ and TGF-β. These data show that the recruitment of T cells into the healing wound is tightly regulated and that the recruited cells produce a suite of inflammatory cytokines with established functions in wound healing. Surprisingly however, skin deficient in CD4^+^ or CD8^+^ T lymphocytes did not exhibit impaired wound closure, collagen content, or angiogenesis, suggesting that T cells are not required for a normal wound healing response in this model, even though depletion of CD8^+^ T cells in wounds reduced but did not abolish the recruitment of macrophages and neutrophils [136].

T lymphocytes are also observed to be recruited early in the healing wound process in a severe combined immunodeficient (SCID) murine model and were predominantly of the CD4^+^ subtype [137]. However, in this case, T- and B-cell deficiency during cutaneous wound healing in SCID mice caused fibrotic scarring, elevated leucocyte and macrophage infiltration, and little neo-angiogenesis, but nevertheless accelerated healing compared with wildtype controls. Restoration of CD4^+^ (but not CD4^−^ and Treg) cells could restore wound healing [137]. These contrasting observations suggest a complex role for subsets of T cells in wound healing via modulation of other immune cell types and it is still unclear whether this involves modulation of the ECM.

### T cells in immune regulation

#### Regulatory T lymphocytes

The majority of CD4^+^ T cells that accumulate after wounding are regulatory T lymphocytes (Treg) [138]. These cells are recruited from secondary lymphoid organs and are highly activated phenotype. The major function of Tregs in skin wounding is to limit the accumulation of IFN-γ producing T cells and pro-inflammatory macrophages in the wounded skin [138]. FoxP3-expressing regulatory T cells (Treg) are essential for tissue immune homoeostasis. The EGFR, well known to be expressed on epidermal cells, is also expressed on Tregs as is the EGFR ligand, amphiregulin (AREG). In full-thickness wounds, Tregs accumulate in skin, and limit IFN-γ production and M1 macrophage accumulation [138]. Depletion of Tregs ‘early’ in wound healing caused greater attenuation of wound healing than when depletion was carried out later in the process, suggesting a role for Tregs in regulating the early immune response of wound healing. Direct roles for Tregs in ECM-mediated regulation of the immune response remains to be uncovered.

#### T cells and fibroblast activation and consequences for ECM homoeostasis

T cells can produce heparin-binding epidermal growth factor-like growth factor (HB-EGF) and basic FGF (bFGF), factors that stimulate fibroblast proliferation and activation [139]. bFGF is synthesised by CD4^+^ and CD8^+^ T lymphocytes, whereas HB-EGF is primarily produced by CD4^+^ T lymphocytes. CD4^+^ TH2 cells produce IL-13 and TAMPS to stimulate collagen synthesis [140], whereas TH1 cells release IFN-γ to degrade collagen [141] indirectly via the action of fibroblasts.

TH2 cytokines include IL-4, IL-5, IL-9, and IL-13, and many have established roles in ECM regulation. In skin, IL-13 can drive myofibroblast activation and increase collagen production [142]. Additionally, fibroblasts express receptors for IL-4 and it has also been shown to stimulate collagen production [143], suggesting that TH1 cells may also be able to stimulate the production of ECM in this way. IL-13 has been shown to induce POSTN production in asthmatic airway [144], further suggesting that T lymphocytes may regulate ECM production under certain pathologic conditions by activating fibroblasts within the local microenvironment.

#### T cells and ECM regulation in pathology

Although T cells do not play a critical role in wound healing, they are utilised in settings of infection and inflammation during healing. In these cases, T cells have the ability to actively remodel the ECM to aid in interstitial migration.

Activated CD4^+^ T cells can express mRNA for MMP-9, MT1-MMP, MT4-MMP (also designated MMP-17), ADAM-9, ADAM-15, ADAM-17, cathepsin L, urokinase-type plasminogen activator (uPA), TIMP-2, PAI-1, but local collagen lysis was not a driver of migration [145]. T-cell migration does not require the activity of proteases, but rather T cells use an amoeboid movement mechanism, driven by adaptive morphology permitting crawling along and between collagen [145] and fibronectin fibrils [146], via an αv-integrin-dependent mechanism [147] and involves the physical deformation of the ECM structure by the exertion of mechanical force.

#### Tumour-infiltrating lymphocytes

Treg cells have the most defined role in cutaneous wound healing, in which they facilitate wound re-epithelialisation by suppression of inflammation responses. In a similar manner, tumour Tregs accumulate in the tumour stroma and hinder anti-tumour immunity [148]. Although the CD4^+^FoxP3^+^ Treg subset of CD4^+^ cells are a significant portion, CD4^+^FoxP3^−^ T helper subset of T lymphocytes also have significant pro-tumour activity. Additionally, CD8^+^ cytotoxic cells are also significant anti-tumour actors. The literature on T-cell immunity in cancer is vast, and a full discussion is outside the scope of this review. Only those studies relevant to ECM interactions will be discussed in this section.

In the context of ECM and immune cells, we highlight that ECM has a significant impact on T-cell accessibility to the tumour cells for their function. Overall, while the infiltration of T cells into solid tumours can correlate with positive prognosis, there are many factors that determine T-cell function within the tumour. The TME can impact the ability of T cells to enter the tumour, but also can have an effect on the cytotoxic activity of the lymphocytes within the tumour [149].

The distribution of T cells in the tumour environment could be guided by the presence of chemoattractants (such as CCL5) in the stroma, rather than the tumour cell regions. The composition of the stroma ECM can have a significant impact on the ability of the lymphocytes to enter the tumour mass. The density of the collagen in ECM can inhibit T-cell locomotion and their stimulation by cytokines cannot overcome this barrier [150]. Examination of human lung tumours determined that T lymphocytes prefer migration in tumour stroma regions containing low levels of FN and collagen [151], consistent with the observed ability of dense ECM to trap these cells.

The effect of collagen on T cells may not only influence migratory potential, but also tumour-infiltrating lymphocyte (TIL) activity. Collagens are the functional ligands for the immune inhibitory receptor LAIR1/CD305 [152]. T cells express LAIR-1 and cross-linking to the collagen ligand can inhibit cellular TCR-mediated function [153]. This effect of LAIR-1 immuno-suppression on CD8^+^ T cells potentiates PD-1/PD-L1 blockade resistance in lung tumours in mice [154], demonstrating a key mechanism by which ECM biology impacts upon the ability of the immune system to modulate tumour growth, with implications for immune therapy.

## Tumour immunity

We have described so far how the regulation of the immune system during cutaneous wound healing begins with pro-inflammatory signals that potentiate the influx of immune cells that fight microbial invasion, but also commence the process of activating fibroblasts, stimulate proliferation of keratinocytes, and promote angiogenesis. Anti-inflammatory signals are then engaged to re-establish homoeostasis, with the restoration and remodelling of the ECM and completion of re-epithelialisation.

During cancer progression, the cellular interactions can exhibit these same pathways may become tumour suppressive or tumour promoting. TANs, TAMs, and TILs all participate in the establishment of the TME by engaging cellular process aligned to their functions in wound healing. An emerging concept is that immune cells, CAFs, and the ECM are regulated in a co-ordinated fashion downstream of signals from the tumour, with CAFs taking a lead role in the *in situ* differentiation of key components of the microenvironment [6].

## Concluding remarks

In this review, we have discussed key aspects of the ECM and the tumour immune system that determine the course of the disease. What is clear is that cancers are able to co-opt the normal functions of immune cells such as those important for wound healing, as well as the ECM into the process of tumorigenesis and tumour progression, although these are modified by the pathological characteristics of the disease. Nevertheless, such divergences in the behaviour of genetically normal cells associated with tumours are key to uncovering the next generation of cancer targets and biomarkers to assist in managing this disease.

## Data Availability

No datasets have been used in producing this work.

## Abbreviations

αSMA: alpha smooth muscle actin
bFGF: basic FGF
CAF: cancer-associated fibroblast
CAR: chimeric antigen receptor
CRC: colorectal cancer
DAMP: damage-associated molecular pattern
ECM: extracellular matrix
ELN: elastin
ERα: estrogen receptor alpha
ERC: elastin receptor complex
FN: fibronectin
GAG: glycosaminoglycan
HB-EGF: heparin-binding epidermal growth factor-like growth factor
HGF: hepatocyte growth factor
IFN: interferon
IL: interleukin
LAIR1/CD305: leucocyte-associated immunoglobulin-like receptor
LTB4: leukotriene B4
MAPK: mitogen-activated protein kinase
MC: mast cell
MMP: matrix metalloproteinase
MT1-MMP: membrane type 1-matrix metalloproteinase
NET: neutrophil extracellular trap
OSCC: oral squamous cell carcinoma
PD-1: programmed cell death protein 1
PD-L1: programmed cell death ligand 1
PDGFRα: platelet-derived growth factor receptor alpha
PI3K: phosphoinositide 3-kinase
POSTN: periostin
ROCK: Rho-associated kinase
SCF: stem cell factor
SCID: severe combined immunodeficiency
SPP1: secreted phosphoprotein 1
TAM: tumour-associated macrophage
TAMP: tumour-associated molecular patterns
TAN: tumour-associated neutrophil
TGF: transforming growth factor
TME: tumour microenvironment
TIL: tumour-infiltrating lymphocyte
TIMP: tissue inhibitor of metalloproteinase
TNC: tenascin-C
TNF: tumour necrosis factor
Treg: regulatory T lymphocyte/cell
TSP: thrombospondin
VEGF: vascular endothelial growth factor
YAP: Yes-associated protein
